# T7. PHARMACOGENETIC OF TARDIVE DYSKINESIA -- A FOLLOW-UP ON THE VALBENAZINE TARGET VMAT2/SLC18A2

**DOI:** 10.1093/schbul/sby016.283

**Published:** 2018-04-01

**Authors:** Clement Zai, Arun Tiwari, Daniel Mueller, Aristotle Voineskos, Steven G Potkin, Jeffrey Lieberman, Herbert Meltzer, Gary Remington, James Kennedy

**Affiliations:** 1 Centre for Addiction and Mental Health; 2 University of California, Irvine; 3 Columbia University; 4 Northwestern University Feinberg School of Medicine

## Abstract

**Background:**

Tardive dyskinesia (TD) is a motor side effect that may arise after long-term treatment of antipsychotic drugs. Its etiology is not well understood, but a number of risk factors have been associated with TD. TD occurrence appears to be familial, thus suggesting a genetic component. We previously reported on an association between the SLC18A2 gene that codes for the vesicular monoamine transporter 2 (VMAT2) that packages monoamines including dopamine from the cytoplasm into synaptic vesicles (Zai et al, 2013). In the present study, we examined the dopamine transporter gene SLC6A3 by itself and in conjunction with SLC18A2 for possible association with TD.

**Methods:**

We genotyped and analyzed the variable-number tandem repeat (VNTR) polymorphism in the 3’ untranslated region of the SLC6A3 gene in our European sample of 187 schizophrenia/schizoaffective disorder patients assessed for TD occurrence based on the Abnormal Involuntary Movement Scale (AIMS). We also explored the interaction between the VNTR and the TD-associated SLC18A2 marker rs363224.

**Results:**

Our preliminary analysis did not show the SLC6A3 VNTR to be associated with TD occurrence or severity. There also appeared to be no significant interaction between SLC6A3 VNTR and SLC18A2 rs363224 in TD occurrence or severity (p>0.05).

**Discussion:**

Our findings did not support a major role of the dopamine transporter gene in TD risk or severity, but we will examine additional putative functional markers in this gene.

